# Content quality versus sharing practices on social media: a cross-sectional analysis of nutrition information on Twitter

**DOI:** 10.1017/S1368980025000461

**Published:** 2025-04-10

**Authors:** Cassandra H Ellis, Peter Ho, J Bernadette Moore, Charlotte EL Evans

**Affiliations:** 1 School of Food Science and Nutrition, University of Leeds, Leeds LS2 9JT, UK; 2 The Nutrition Society, 10 Cambridge Court, 210 Shepherds Bush Road, London W6 7NJ, UK

**Keywords:** Nutrition communication, Quality assessment, Digital health, Online information, Social media, Twitter, X

## Abstract

**Objective:**

To use the validated Online Quality Assessment Tool (OQAT) to assess the quality of online nutrition information.

**Setting:**

The social networking platform was formerly known as Twitter (now X).

**Design:**

Utilising the Twitter search application programming interface (API; v1·1), all tweets that included the word ‘nutrition’, along with associated metadata, were collected on seven randomly selected days in 2021. Tweets were screened, those without a URL were removed and the remainder were grouped on retweet status. Articles (shared via URL) were assessed using the OQAT, and quality levels were assigned (low, satisfactory, high). Mean differences between retweeted and non-retweeted data were assessed by the Mann–Whitney *U* test. The Cochran–Mantel–Haenszel test was used to compare information quality by source.

**Results:**

In total, 10 573 URL were collected from 18 230 tweets. After screening for relevance, 1005 articles were assessed (9568 were out of scope) sourced from professional blogs (*n* 354), news outlets (*n* 213), companies (*n* 166), personal blogs (*n* 120), NGO (*n* 60), magazines (*n* 55), universities (*n* 19) and government (*n* 18). Rasch measures indicated the quality levels: 0–3·48, poor, 3·49–6·3, satisfactory and 6·4–10, high quality. Personal and company-authored blogs were more likely to rank as poor quality. There was a significant difference in the quality of retweeted (*n* 267, sum of rank, 461·6) and non-retweeted articles (*n* 738, sum of rank, 518·0), U = 87 475, *P*= 0·006 but no significant effect of information source on quality.

**Conclusions:**

Lower-quality nutrition articles were more likely to be retweeted. Caution is required when using or sharing articles, particularly from companies and personal blogs, which tend to be lower-quality sources of nutritional information.

It is becoming increasingly common for the public to turn to the internet and social media sources for nutrition information^(1)^. However, the digital environment has minimal regulation and varying quality^(2)^, which increases the risk of exposure to misinformation^(3)^ and knowledge distortion^(4)^. To add to the complexity, social media facilitates rapid dissemination of content^(5)^, allowing myths to spread quickly^(6)^ potentially creating an environment where ‘often the loudest, most extreme voices drown out the well informed’^(7)^.

In recent years, there has been a proliferation of professional bloggers giving lifestyle and dietary advice^(8,9)^. In the context of nutrition, many bloggers have thousands of followers but no relevant nutritional science qualifications^(6)^. Indeed, it has been found that only 6 % of American food bloggers have nutrition degrees^(10)^. This type of non-expert-generated content may explain the variation in the quality of the digital environment. For example, healthy eating blogs from credentialed experts were found to be of higher quality in comparison to non-experts, with 43 % of all blogs reviewed aligning with dietary advice^(9)^. Similarly, articles on COVID-19 and vitamin D are inconsistent with the scientific evidence^(11)^, and articles giving information on vegan diets are varied and unreliable^(2)^.

Supporting these results, personal and commercial blogs^(12)^ have been found to be consistently of poorer quality than other sources of online information^(2,13)^, providing lifestyle and nutrition advice that is subjective and unbalanced^(14)^. In part, this could be explained by coverage of the UK Article 12(c) on Nutrition and Health Claims Regulation^(15)^. Although this regulation prohibits health professionals from discussing certified health claims in commercial communications, non-professionals, celebrities and ‘influencers’ do not fall under this regulation and can discuss health claims, whether certified or not^(16)^.

Similar patterns of poor-quality nutrition information being disseminated by non-expert bloggers have been evidenced on social media^(17)^. A study using Instagram found that weight management posts by social media influencers were to be of poor quality^(18)^. The ‘healthy diet’ discourse on Twitter has been found to be dominated by ‘non-health professionals’ and largely constitutes poor quality information that contradicts public health advice^(5)^. Beyond just quality, examining social media can provide unique insights into the nutrition and diet information reaching, and influencing, large segments of the general population^(19)^. In addition, it is important to understand sharing practices as Twitter posts are also subject to likes and retweets. Previous research has investigated emotion as a motivator for retweeting news^(20)^, but to our knowledge, information quality, and whether quality is a predictor of engagement, has not been investigated. Therefore, in the context of the widespread sharing of misinformation, it is important to understand the quality of the information that has the potential to be widely shared and how this influences the debate in question^(21)^.

Nutrition research is at particular risk of misunderstanding as people have daily interactions with food, and beliefs may be rooted in cultural practices, assumption and intuition, more than sound science^(22)^. Prolonged exposure to inconsistent nutrition information over a period of time can have detrimental effects on consumer beliefs^(7,23)^ and impact adherence to recommended nutrition behaviours such as fruit and vegetable consumption^(24)^. Therefore, it is increasingly important to be able to differentiate between high- and low-quality nutrition information and determine the sharing practices of different types of information. However, to date, it has been difficult to compare the quality across existing studies due to their use of multiple quality criteria and different assessment tools. Notably, Afful-Dadzie and colleagues examined the quality of health information shared on online and found that most of the literature relied on three quality assessment tools^(25)^. Afful-Dadzie et al concluded these tools were outdated and not fit for purpose; moreover, they called for standardised quality assessment criteria suitable for social media and online content. In response to this, we have developed and validated a novel quality assessment tool, specifically suited to assessing the quality of online nutrition information^(13)^.

The current study uses the aforementioned newly developed assessment tool to address a further gap in the literature, namely to assess the quality of online nutrition information disseminated via Uniform Resource Locators^(26)^ via Twitter. Twitter was of interest in this study as it remains a popular platform for discussing news and nutrition-related information. A crucial function of Twitter as a platform is information sharing^(26)^, including URL to external articles which is active and demonstrates engagement with content. Twitter also allows second-degree sharing, or retweeting, giving a further indication of the content the public is engaging with. Therefore, we specifically aimed to examine the quality of retweeted articles, shared via URL, in comparison to unshared content, in order to determine: (1) whether the high- or low-quality information is more likely to be retweeted and (2) which information sources were sharing the highest quality nutrition information.

## Methods

Using our previously validated tool designed to measure the quality of online nutrition information^(13)^, we aimed to analyse the quality of a randomly selected subset of nutrition-related articles posted via URL on Twitter in 2021. While Twitter changed its name to X in July 2023, the data collected for this study were collected from Twitter; therefore, we will continue to refer to the platform as Twitter and use the terms tweets and retweets throughout.

### Data collection and screening

The Twitter Search application programming interface (API), as it was known before the rebrand to X, was used to gather data. The dataset comprised all English language tweets including the word ‘nutrition’ by month from 1 January 2021 to 31 December 2021. A full year was collected to allow a random sample from across the year to be analysed which would not be affected by any predetermined seasonal effects, usually seen in December and January^(27)^.

The tweets themselves were out of scope in this study as the OQAT was designed to measure the quality of longer-form online articles written to give dietary and nutrition advice to the public. Similarly, because of the character restrictions of Twitter, the posts themselves are unlikely to score high on the OQAT criteria. Instead, Twitter was used to 1) collect articles (shared via URL) that the public have interacted with at least once (through the initial act of posting) for the quality assessment and 2) assess the type of online article that the public are engaging with and whether the quality was a factor in the decision to reshare articles.

Using www.random.org, 4 days were selected for analysis, 24 January, 11 August, 21 November and 22 November 2021. There were more tweets collected that had not been retweeted, therefore three additional days were randomly selected: 26 May, 12 June and 14 December 2021, and the retweeted tweets were included for analysis. This gave approximately the same number of URL in each category (retweet and no retweet) before screening for relevance. The data were then filtered by those containing a URL, and tweets that did not include a URL were discounted. This established two datasets: URL with and without retweets.

Each eligible article (shared via URL) was reviewed manually and categorised based on the Online Quality Assessment Tool (OQAT) codebook^(13)^ to identify the website source and the content type. The URLs were included if they were related to human health and discussed any of the following: diet and disease risk, diet and disease management, nutrition and dietary advice, scientific research papers relating to human nutrition, or, specific macro or micronutrients. Articles were excluded if topically irrelevant, linked to social media or consisted of advertising and product promotion. Articles that related to climate change, animal nutrition, food and agricultural policy were discounted if they did not directly relate to nutrition and human health. In addition, articles were discounted if they were part of discussion forums, videos or linked to other social media accounts as the OQAT was only designed to measure written information. Finally, scientific research papers were also excluded. This was because research papers are not necessarily intended to be public facing or to give dietary advice and therefore have less direct impact on dietary choices. Additionally, when we developed the OQAT and carried out pilot testing, scientific studies scored 9/10 (noting they do not include expert quotes) therefore this could have skewed the results; however, press releases were included as the public-facing aspect of scientific papers.

Two trained raters used the tool independently to score the relevant articles against the ten OQAT indicators. The indicators were designed to measure three criteria: (1) Currency: publication date, author name and credentials; (2) Credibility; links to high-quality references, specialist quote, transparency and (3) Reliability; adequate background, reflective headline, does not over generalise, does not have potential to cause undue harm or optimism. Indicators were scored positively, and an article could score between the values of 0 and 10. A higher OQAT score indicated a higher quality article. During previous validation, the OQAT had moderate internal consistency (*α* = 0·382). Cohen’s Kappa coefficient demonstrated high interrater agreement (k = 0·653, *P*< 0·001). Full details on the development of the criteria and indicators can be found in the published validation report^(13)^.

Any discrepancies were discussed among raters until a consensus was reached. After scoring, articles were ranked into three categories using the OQAT measure obtained from the Rasch analysis described in the next section. The source of the article was also recorded by the OQAT. Articles were manually categorised by raters and categorised as one of the following 10 sources: (1) Blog – personal, (2) Blog – professional, (3) Company, (4) Government organisation, (5) Magazine, (6) Non-Governmental Organisation (NGO), (7) Professional news, (8) Research institute/University/publisher, (9) social media (out of scope) and (10) unrelated (out of scope). Raters met to discuss and agree on any ambiguity. Rater one checked a random sample of rater two’s scores to ensure the correct application of the OQAT, any discrepancies were discussed and agreed. Rater reliability was checked using the Rasch model; results are presented in the online supplementary material, Supplemental material.

### Statistical analysis

The Statistical Package for the Social Sciences (SPSS v 28.0) was used for statistical analysis, and the R computing environment (v 4.2.3) was used for data visualisation. After all tweets including the word ‘nutrition’ posted in 2021 were collected, tweets were collated and those including a URL were identified. The raw data were charted to visualise the annual data collection. The data collection and screening were visualised in a flowchart. Descriptive statistics were reported including total scores, medians and interquartile ranges, which were calculated for each media source and by retweet.

A total measure for evaluating quality was obtained by fitting the Rasch dichotomous model to the ten-item OQAT questionnaire using Winsteps (v5.3.2.0). The Rasch model has been applied in many disciplines^(28,29)^ and is intended for the examination of measurement instruments such as the OQAT. Rasch outfit mean squared errors of 0·5–1·5 were used to determine the adequate fit of items to the Rasch model. In this study, Rasch allowed for a single interval scaled measure that represented the underlying construct of quality, as measured from ten question items (the quality measure) without the need to assign weight in advance. Therefore, quality levels (low, satisfactory, high) were established by determining statistically significant levels in the Rasch measures based on the procedures suggested by Wright^(30)^. Prior to determining the quality levels, interrater reliability was also examined with a separate Rasch model, to confirm that data could be combined in a single analysis.

As the data were categorical, the Cochran–Mantel–Haenszel test^(31)^ was used to examine the associations between high, satisfactory and low-quality articles, and whether they were more or less likely to be retweeted. The contingency analysis is displayed as a Fourfold graph to allow the categorical data to be visualised^(32)^. The Woolf test^(33)^ was used to test the homogeneity between log odds ratios in each strata to determine whether the Cochran–Mantel–Haenszel test was valid. Cochran–Mantel–Haenszel test was also used to investigate whether there was a significant difference between sources when comparing whether they were retweeted. Cochran–Mantel–Haenszel test was chosen as it is more robust when some of the strata contain small frequencies. After the contingency and chi-square analysis, articles were manually reviewed by rater one to see whether it was possible to infer any rationale for differences between groups.

The Shapiro–Wilks test was used for the normality of retweets and non-retweeted data indicating that the data were not normally distributed (*P*< 0·001). The natural logarithm was used to transform the data but did not rectify the distribution and therefore non-parametric tests were used to compare tweets and retweets. The Mann–Whitney *U* test was used to analyse any differences in rank scores of retweeted and unshared data.

## Results

### Data collection

**Figure 1.**
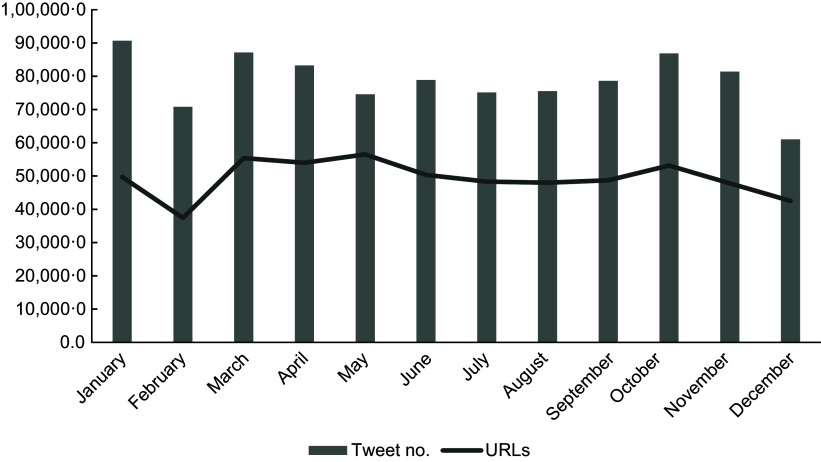
Total number of tweets categorised as nutrition information and URLs collected by month in 2021.

Over the full 12-month collection period, 943 869 tweets were collected, and of these 591 907 contained an URL (Figure 1).

**Figure 2.**
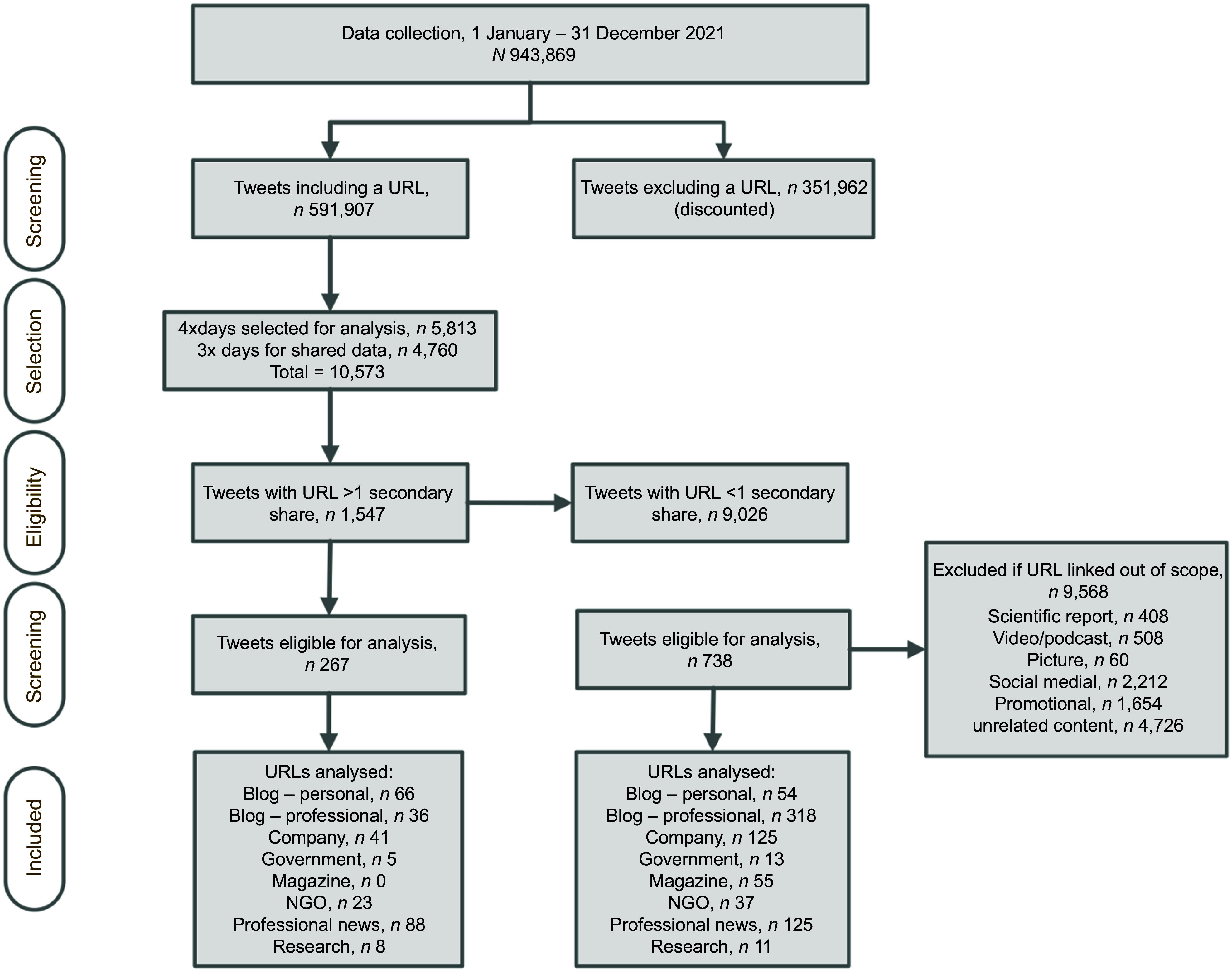
Flow diagram of identification and screening of tweets for analysis to assess the quality of online nutrition information.

During the analysis period, 10 573 URL were collected from 18 230 Twitter posts. After manual screening for relevance, these represented professional blogs *n* 354 (35·2 %), news outlets *n* 213 (21·2 %), companies *n* 166 (16·5 %), personal blogs *n* 120 (11·9 %), NGOs *n* 60 (6·0 %), magazines *n* 55 (5·5 %), research institutes or publishers *n* 19 (1·9 %), government organisations *n* 18 (1·8 %), 9568 articles were excluded as they were out of scope (Figure 2).

### Fit and inter-rater analysis

Rasch analysis was conducted to ensure the OQAT criteria and indicators measured what they were designed to measure and check inter-rater reliability. The Rasch analysis of the data indicated that all ten items complied with the recommended OUTFIT mean squares between 0·5 and 1·5 for being ‘productive for measurement’^(30)^. Rasch analysis also confirmed that all sources met indicator 9, and that indicators 4, 5 and 6 were necessary for an article to be classified as high. Figure three shows the fit with outliers removed for Q6 and Q9 to improve fit. Removing the outliers improved the fit but did not change the conclusions.

**Figure 3.**
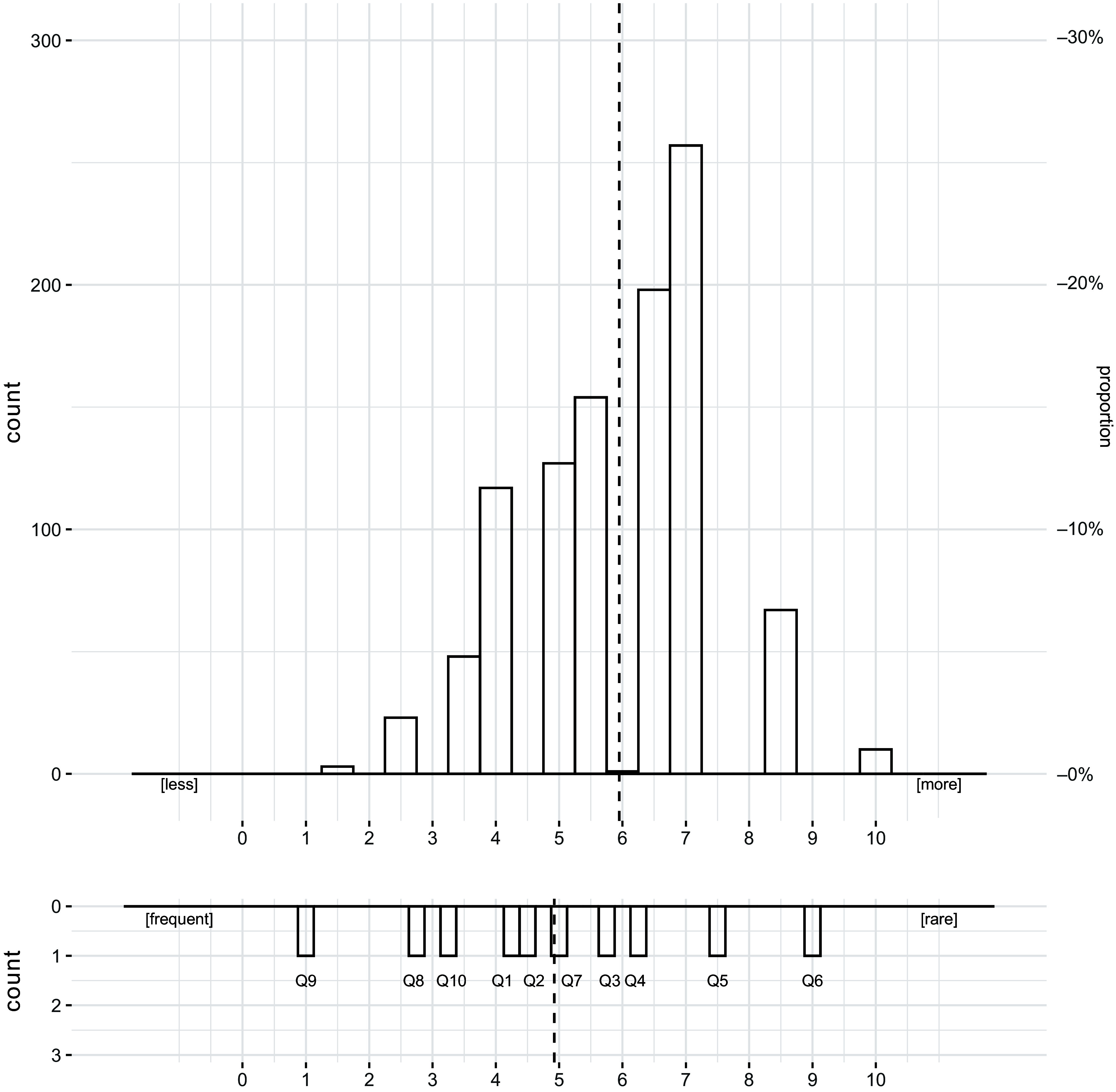
Wright map illustrating the quality of each article and discriminating quality assessment indicators. The upper plot shows the quality of each article. The lower plot illustrates the fit of all quality assessment indicators. Shaded areas from left to right of the plot correspond to increasing levels of quality (low, satisfactory, high). All estimates were rescaled from 1 to 10. The dotted line represents the mean score.

The Wright map (Figure 3) shows the indicators (Q1–Q10) ranked by prevalence, left to right. Details of what these indicators are designed to measure can be found in the author’s previous paper^(13)^. The lower plot indicates the indicator (Q1–10) by order of prevalence, left to right. Indicators on the left were more likely to be scored positively in the articles than those on the right. Therefore, we can see that all articles scored positively on Q9, and the least likely criterion to be met was Q6. The shading on the lower plot indicates the quality rank (low, satisfactory and high quality), and therefore, we are able to determine that Q9, Q8 and Q10 were necessary for an article to score 3 and be deemed low quality, Q1, Q2, Q7 and Q3 were necessary for articles to be classified as satisfactory and Q5 and Q6 were required for an article to be high quality.

To ensure inter-rater consistency, the Rasch model was used to compare the two independent sets of rater scores. The distribution confirms that the value added to each criterion by each rater is the same inferring consistency between rater (see online supplementary material, Supplemental 1).

### Descriptive analysis

To assess quality, articles were categorised as poor, satisfactory and high quality based on the OQAT measure; 0–3·48 indicated poor quality, 3·49–6·3 indicated satisfactory quality and 6·4–10 indicated high quality. The quality levels are as identified by the OQAT using Rasch analysis which identified the minimum requirements for each category^(13)^.

The relevant articles (*n* 1005) were assessed using the OQAT. As per the OQAT guidelines, 33 % (*n* 335) of articles were categorised as high quality, 59 % (*n* 595) as satisfactory and 7 % (*n* 74) were defined as poor quality articles (Table 1).

**Table 1.** Online quality assessment tool ranked by shared status, content type and media source

		Shared (Y/N)	*n*	%	Median	IQR	Poor	%	Satisfactory	%	High	%
Total (*n* 1005)		Yes	267	26·57	5·5	1·43	25	9·4	176	65·9	66	24·9
		No	738	73·43	6·3	2·37	49	6·6	419	56·8	270	36·6
		Total	1005	100	7·0	3	74	7·4	595	59·2	336	33·4
Media Type	Blog – Personal	Yes	66	55·0	4·87	1·35	12	18·2	47	71·2	7	10·6
		No	54	45·0	5·5	2·09	7	13·0	37	68·5	10	18·5
		Total	120	12·0	5·55	2·10	19	15·8	84	70·0	17	14·2
	Blog – Professional	Yes	36	10·2	6·3	1·26	1	2·8	28	77·8	7	19·4
		No	318	89·9	6·3	2·37	23	7·2	162	50·9	133	41·8
		Total	354	35·2	6·3	2·37	24	6·8	190	53·7	140	39·5
	Company	Yes	41	24·7	4·87	1·72	7	7·1	30	73·2	4	9·8
		No	125	75·3	4·87	2·10	16	12·8	92	73·6	17	13·6
		Total	166	16·6	4·87	1·10	23	13·9	122	73·5	21	12·7
	Government	Yes	5	27·8	5·55	2·60	1	20·0	3	60·0	1	20·0
		No	13	72·2	4·87	1·39	1	7·70	11	84·6	1	7·7
		Total	18	1·8	4·87	1·60	2	11·1	14	77·8	2	11·1
	NGO	Yes	23	38·3	6·3	1·69	1	4·3	15	65·2	7	30·4
		No	37	61·7	5·55	1·43	2	5·4	30	81·1	5	13·5
		Total	60	6·0	5·55	1·43	3	5·0	45	75·0	12	20·0
	News	Yes	88	41·3	6·3	0·94	3	3·4	48	54·4	37	42·0
		No	125	58·7	7·24	0·94	–		53	42·4	72	57·6
		Total	213	21·2	7·24	0·94	3	1·4	101	47·4	109	51·2
	Magazine	Yes	–	–	–	–	–		–		–	
		No	55	100	6·30	1·69	–		28	50·9	27	49·1
		Total	55	5·5	6·3	1·69	–		28	50·9	27	49·1
	Research Institute	Yes	8	42·1	6·3	3·15	–		5	62·5	3	37·5
		No	11	58·9	6·3	1·69	–		6	54·4	5	45·5
		Total	19	1·9	6·3	1·69	–		11	57·9	8	42·1

### Retweet and no retweet comparison

Articles that were not retweeted (*n* 738, mean = 6·03) scored higher on the OQAT than those that had been retweeted (*n* 267, mean = 5·731). There was a significant difference in the quality of retweeted (*n* 267, sum of rank, 461·62) and non-shared data (*n* 738, sum of rank, 517·97), U = 87 475, *P*= 0·006. Articles categorised as poor and satisfactory by the OQAT, with a score of < 6·3, were more likely to be retweeted. Similarly, articles defined as high quality had fewer retweets.

### Media source

The media source of the article was recorded by the OQAT. The Woolf test was used to test homogeneity of the logs ratio for each strata to ensure the Cochran–Mantel–Haenszel test assumptions were met and it was the most appropriate test, *P*= 0·853. The mean scores for each media source were calculated with professional news outlets having the highest score, mean = 6·67, and company blogs the lowest, mean = 5·11 (Table 1). When comparing retweeted and unshared by source, news had the highest mean score (retweeted 6·47, unshared 6·42); however, personal blogs had the lowest retweeted mean (4·92) and company blogs not retweeted had the lowest mean (5·14).

### Quality by media source

**Figure 4.**
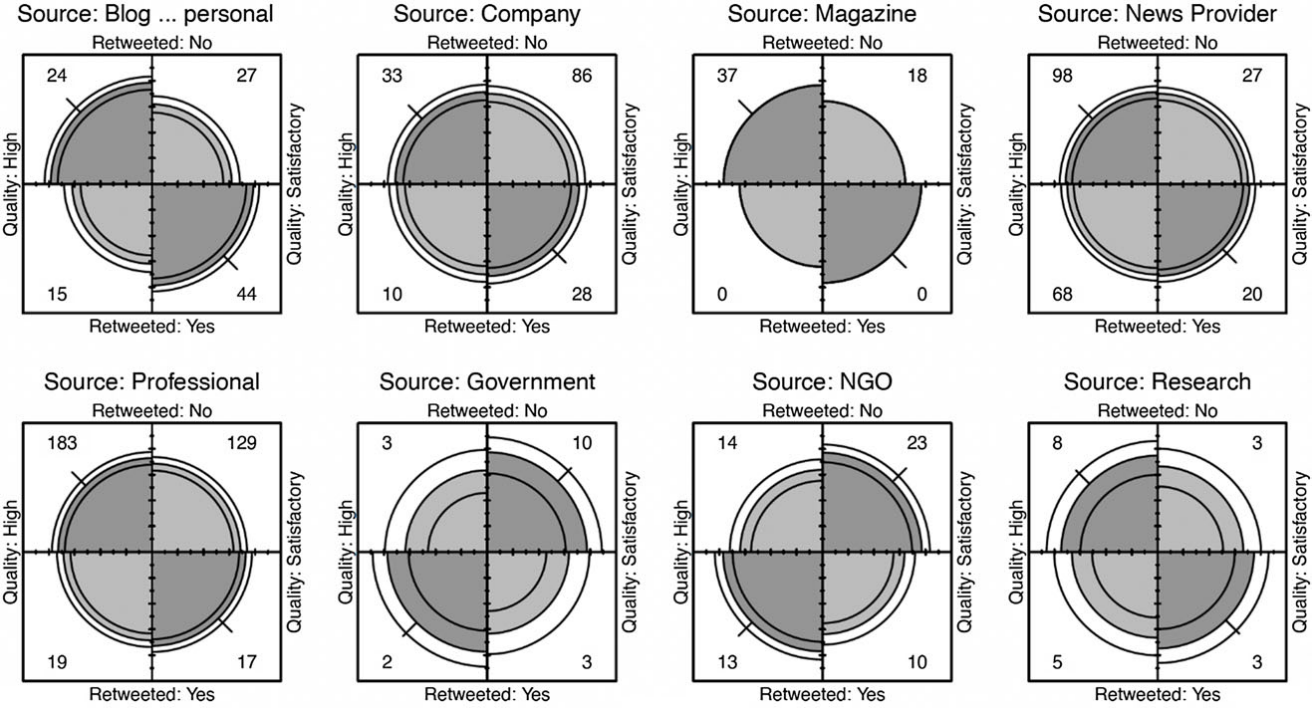
Fourfold display of article quality (High *v* Satisfactory) by source. In each panel, the darker shaded diagonal areas with greater area than the off-diagonal areas show a positive association. The confidence rings for adjacent quadrants overlap if the OR for quality and retweet does not differ significantly from 1.

The Cochran–Mantel–Haenszel test was used to investigate whether there was a significant difference between sources when comparing whether they were retweeted. Results comparing high and satisfactory articles are displayed in Figure 4. When analysing the source quality, a comparison of high- and low-quality articles was also carried out (not presented) but because the group sizes of the low-quality articles were small, there was no significant difference, *X* ^2^_MH_ = 1·2487, df = 1, *P*= 0·264. Similarly, there was no significant difference between satisfactory and low, *X* ^2^_MH_ = 0·017, df = 1, *P*= 0·898 or between high and satisfactory, *X* ^2^_MH_ = 0·888, df = 1, *P*= 0·346 (Figure 4). The low numbers of retweets in most groups may have influenced the significance. Figure 4 shows the differences within groups; for example, the government and research categories had low numbers of retweeted articles.

## Discussion

In this study, we measured the quality of a representative subset of public-facing online nutrition information using a validated tool designed specifically for nutrition research, addressing an important gap in the literature. Importantly, we investigated whether Twitter users were more likely to retweet high- or poor-quality information and which media sources were more likely to share higher quality nutrition information. Our results show, for the first time, a significant difference in the quality of retweeted and non-retweeted nutrition articles, with lower-quality content more likely to be retweeted.

There remains a paucity in the nutrition literature on whether the quality of information is a predictor of sharing, although poorer quality videos have been found to have more views and likes^(34)^. Additionally, the lack of evidence-based information retweeted in our study was consistent with the literature pertaining to anti-climate change blogs^(35)^ and public authored political blogs^(36)^. In this study, higher-quality nutrition sources were less likely to be retweeted. Indeed, articles defined as poor or satisfactory were more likely to be retweeted. This suggests that either quality is not an important consideration for Twitter users when choosing to retweet, or that people are generally unable to discriminate between high- and low-quality nutrition information. As articles ranked satisfactory were the most retweeted, further investigation was carried out into whether users were more likely to retweet articles scoring high or low within the satisfactory range. There was not enough evidence to determine whether quality was a factor affecting retweet decisions for these users.

Analysis of climate change content shared on Twitter found that the accuracy of content does not impact sharing, rather novel content was more likely to be shared and retweeted^(37)^. There is also another possibility, in that people do not read articles before sharing and therefore are not able to make an informed decisions on quality^(38)^. However, in this study, more satisfactory than low-quality articles were retweeted in this study suggesting that some quick ‘sense checks’ of quality may be taken before sharing. This aligns with previous research which suggests that some members of the public do engage in rapid checks to validate online health information before sharing^(39)^.

Article quality varied greatly however poorer quality information was more likely to be retweeted than high quality. This supports previous work whereby online blogs scored poorly when measured against dietary advice. In particular, content which scored poorly was less likely to provide references to scientific evidence, provide expert quotes or declare any author conflicts of interest^(2,9,18)^. When comparing the quality of articles by source, the group sizes were not equal so calculating effect size was not possible. There appears to be a relationship between the source and the quality of the article, with commercial websites scoring lower, and professional news outlets scoring higher. This is supported by the literature. YouTube videos are higher quality when produced by experts,^(34)^ and lifestyle websites written by commercial companies lack objectivity and transparency^(14)^. Similarly, commercial websites giving advice on dietary supplements are more likely to be poor quality than those authored by health experts^(12)^.

Interestingly, our results show magazine articles were unique in that they did not have any retweets, regardless of the quality of the article. In-depth analysis of these articles suggests that magazine articles may be distinctive in that they target a specific cohort such as women, marathon runners or vegans. The language used for magazine articles was simple and they were targeted towards the public; however, they were more likely to give healthy eating advice to a specific group with specific requirements which may not have been novel enough to retweet^(37)^. Articles targeting women may be less likely to be retweeted as fewer women use Twitter compared with men^(40)^. Magazine articles were also more likely to be subscription-based with access limited; therefore, people may be less willing to retweet content that their networks are unable to access.

A further novel finding was that articles shared by government agencies were also less likely to be retweeted than other sources^(41)^, particularly if they were giving public health advice. Articles that related to population health and diets which were written for public health professionals were more likely to be retweeted. This could be due to academics and professionals being encouraged to use Twitter as a medium to disseminate research and network with peers. This could also be because the retweets were from other organisations, and these resources are therefore being used in a professional capacity; however, this level of network analysis was out of scope for this study.

Including scientific references (Q4), quoting a specialist (Q5) and disclosing conflicts of interest or financial interests (Q6) were necessary criteria for articles to be deemed high quality. As shown in the Wright Map (Figure 3), these essential (Q4, Q5, Q6) were the least likely indicators to be achieved. This is consistent with the published literature whereby seeking expert opinion, a sign that the writer was concerned with fact-checking, was lacking in many articles^(42)^. The lack of evidence-based information shared was also consistent with the literature pertaining to print news^(43)^, obesity^(44)^ and dietary advice to cancer survivors^(45)^. All of which highlighted the damage poor quality non-expert written information can have on public health and adherence to dietary guidelines. More encouragingly, the vast majority of articles scored positively on naming an author, an assessment indicator which has previously been shown to positively affect article quality^(42,43)^. In our dataset, this criterion was necessary for an article to be ranked as satisfactory.

At the point of data collection, only five articles had more than five retweets. This was notable as most retweeted articles had just one or two retweets. The most retweeted post was a high-quality article originally published by the World Food Programme, and originally tweeted by António Guterres, Secretary General of the United Nations. The next highest retweeted post was a link to a satisfactory article posted by a high-profile Twitter user with 4 million followers. Both of these Twitter users have large networks suggesting that the user network could be more influential than the quality of the article; however, as networks were not investigated in this research, we do not have enough information to confirm the influence of Twitter networks.

### Strengths and limitations

The main strength of this study is that it used a validated set of standardised assessment criteria^(13)^, as called for in the literature^(14,25)^, to assess the quality of nutrition information available online and shared on Twitter. By using a tool developed specifically to assess the quality of nutrition-related online content, our findings build upon recent studies that have categorised the positive characteristics of dietitian-authored blogs^(9)^ and compared the quality of the blogs to those from lay authors^(46)^. A further strength is the high inter-rater reliability. In this study, the two raters applied the OQAT consistently when rating the independent set of sources. In addition, to the author’s knowledge, this is one of the only studies to quantify the quality of nutrition information by the source publishing the content.

Our data collection was novel in that it used Twitter as the source of articles (shared via URLs) to objectively select a cross-section of online articles designed to disseminate nutrition information. Therefore, each article analysed was interacted with at least once through the initial tweet reducing the likelihood of collecting passive content which does not stimulate reader engagement^(47)^. Additionally, these articles have increased chances of being viewed by the public as they are in the public domain in at least two formats, on the website and on Twitter. The random selection of days for analysis was a strength as it reduced the risk that the discourse was affected by seasonal variation^(27)^.

However, there are some limitations to this study. The disproportionately lower number of retweeted articles compared with non-retweeted made comparison between groups difficult. Nonetheless, a greater number of nutrition-related articles not being retweeted is in line with the author’s previous research investigating obesity articles online^(44)^ and pilot studies using the OQAT^(13)^. In addition, the differing numbers between the sources limited the comparison between these groups. Future research could also categorise articles differently comparing the quality of the type of content shared and not just the source.

A further limitation of the study methodology was that the raters were not blind to the article source. This could have introduced rater bias and caused the rater to moderate the article score based on subjective opinion. However, the OQAT criteria and indicators were worded as clearly as possible to reduce the risk of this type of bias, and inter-rater reliability was analysed to check that the OQAT was being applied consistently. In addition, only webpages were considered; therefore, the wider limitations of the general website function were not considered. Similarly, this study did not consider article readability, as these can be assessed by external software such as Flesch–Kincaid readability test. Finally, only English-language tweets and articles were included in the data set, so these findings may not be generalisable to tweets in other languages or non-English speaking countries. Approximately 40 % of all tweets are written in English therefore a large proportion of nutrition-related content was not considered in this research and is worthy of further exploration.

Although meta-data was collected, we are not able to infer motivations for retweeting beyond quality or any information about social networks. This is a limitation and an area for further research using social network theory to investigate Twitter networks, what users are sharing and retweeting, and who are the users sharing nutrition information. Similarly, this study did not consider the device users were sharing content on so we are unable to make inferences on whether users are more likely to share content on mobile devices *r*, nor did we consider the feasibility of sharing through ‘share’ buttons on websites. However, future research considering the dissemination of content through networks could consider these factors.

Importantly, this research investigating the quality of information has led to a number of recommendations. Online content remains a popular source of nutrition advice for the public^(9,18)^, but the quality is variable^(44,48,49)^. Our recommendations to authors of online nutrition content are firstly, that to be considered high-quality content, any article providing dietary advice must be evidence-based and include hyperlinks to the evidence or provide references. Secondly, hyperlinks and references must directly cite the evidence and not opinion-based articles self-promoting other content on the same website. As digital content easily allows for hyperlinking content and an increasing proportion of nutritional journals are open access, it is proposed that it is best practice to include scientifically validated weblinks.

In addition, online content has an infinite lifespan and therefore should include a published date and a review date. This was an essential criterion for articles to be considered of satisfactory quality. It is a necessary addition to ensure the reader can make informed decisions on the relevance and quality of the evidence presented and whether it includes out-of-date research. Another criterion required to be considered high quality is to include endorsements from specialists and subject matter experts. Expert quotes act as a mark of quality informing the reader that this is a well-researched article that has been subject to informal peer review. Finally, any funding or conflicts of interest should be explicitly stated for an article to be deemed high quality. This informs the reader of any potential author or publication bias and again allows the reader to make an informed decision on whether the article is trustworthy. Further recommendations are included in the OQAT development and validation paper^(13)^.

These findings demonstrate the essential features necessary for articles to be deemed high-quality. Specifically, including scientific references, quoting a specialist, and transparency. In this research, these indicators were the least likely to be achieved, therefore educating content writers on the importance of including these is essential to improve the quality of information. With further testing, the quality assessment indicators from the OQAT could be employed as a checklist for content writers providing a framework for higher-quality information. Similarly, as the public appears to be more likely to repost poor-quality articles, improving digital health and media literacy could be a beneficial intervention. A simple tool such as the OQAT could have far-reaching benefits for the public if it was applied as a framework for readers to assess the quality of information before reading. Although we caveat that the OQAT use would need to be tested in this cohort before this could be implemented.

Further research should consider using the OQAT on a larger data set with more homogenous groups to test whether the differences observed are significant. The current dataset is limited to one social networking site, Twitter, which does not capture all social media users and represents only one platform for sharing health information. Future research is needed that compares different public sources of nutrition and diet information and different social media platforms. In addition, future research should consider the broader influences on retweeting beyond quality, with consideration given to the influence of the person posting the original tweet, the reach of their social media network and the influence of the site where the article is originally published. Finally, it is important for future research to explore the wider nutrition discourse on social media and the flow of information through networks to understand motivations for sharing nutrition content and the key actors involved.

### Conclusions

The quality assessment of online nutrition information using a validated tool designed specifically for this purpose adds to a body of literature assessing the quality of information in the media and online. This study contributes to the understanding of which sources of information the public are likely to engage with and what factors may motivate them to engage with it.

## Supporting information

Ellis et al. supplementary material Ellis et al. supplementary material

## Supplementary material

For supplementary material accompanying this paper visit https://doi.org/10.1017/S1368980025000461
