# Supplementary material for: Content quality versus sharing practices on social media: a cross-sectional analysis of nutrition information on Twitter
Source: Public Health Nutr. 2025 Apr 10;28(1):e77. doi: 10.1017/S1368980025000461 (PMC12100564; doi:10.1017/S1368980025000461)
Supplement: Ellis et al. supplementary material [file S1368980025000461sup001.docx]

**SUPPLEMENTARY MATERIAL**

Supplemental material for Content quality versus sharing practices on social media: A cross-sectional analysis of nutrition information on Twitter

**Table of Contents**

Figure 1a Wright Map of Rater One scores

Figure 1b Wright Map of Rater Two scores

**Supplement 1 Scores by rater**

**Figure 1a Wright Map of Rater One scores Figure 1b Wright Map of Rater Two scores**

**Rasch model was used to compare the two independent sets of rater scores. The distribution of scores was modelled, both including and removing outliers. Removing the outliers improved the fit, but did not change the conclusions. The distributions confirms that the value added to each indicator by each rater is the same inferring consistency.**
